# DDIT4 regulates mesenchymal stem cell fate by mediating between HIF1α and mTOR signalling

**DOI:** 10.1038/srep36889

**Published:** 2016-11-23

**Authors:** Borzo Gharibi, Mandeep Ghuman, Francis J. Hughes

**Affiliations:** 1 Division of Tissue Engineering and Biophotonics, Dental Institute, King’s College London, Tower Wing, Guy’s Hospital, London, SE1 9RT. UK

## Abstract

Stem cell fate decisions to remain quiescent, self-renew or differentiate are largely governed by the interplay between extracellular signals from the niche and the cell intrinsic signal cascades and transcriptional programs. Here we demonstrate that DNA Damage Inducible Transcript 4 (DDIT4) acts as a link between HIF1*α* and mTOR signalling and regulation of adult stem cell fate. Global gene expression analysis of mesenchymal stem cells (MSC) derived from single clones and live RNA cell sorting showed a direct correlation between *DDIT4* and differentiation potentials of MSC. Loss and gain of function analysis demonstrated that DDIT4 activity is directly linked to regulation of mTOR signalling, expression of pluripotency genes and differentiation. Further we demonstrated that DDIT4 exert these effects down-stream to HIF1α. Our findings provide an insight in regulation of adult stem cells homeostasis by two major pathways with opposing functions to coordinate between states of self-renewal and differentiation.

The regulation of quiescence, self renewal and differentiation is a key factor in stem cell biology. Recent studies suggest that the mechanistic target of rapamycin (mTOR) pathway plays a key role in the regulation of stem cell fate^1^. mTOR signalling has been shown to promote proliferation and differentiation of mesenchymal stem cells (MSC)^2^,^3^,^4^,^5^. However, persistent long term activation of mTOR can also result in premature aging and the depletion of the pool of self-renewing stem cells^6^,^7^,^8^. Inhibition of mTOR has been shown to prevent aging in stem cells of hematopoietic, mesenchymal and epithelial origins^6^,^9^,^10^,^11^,^12^.

The role of mTOR signalling in the regulation of stem cell differentiation and aging suggests that stem cell niches may repress excess mTOR activation in order to maintain stem cell quiescence during homeostasis. In support of this suggestion it is known that a hypoxic microenvironment, an important component of HSC and MSC niches, is able to inhibit mTOR through multiple pathways^13^. Similarly, mTOR is regulated in response to metabolic cues which have also been shown to maintain stem cell function during aging^14^,^15^. However the factors that may link these environmental cues with determination of cell fate are not fully understood. Amongst the known upstream repressors of mTOR, the protein DNA-Damage-Inducible Transcript 4 (DDIT4) (also known as Redd1, RTP801) inhibits mTOR in response to both hypoxia and nutrient restriction^16^,^17^.

Here we propose DDIT4 as a response element that link the environmental cues such as hypoxia to mTOR signalling and regulation of MSC fate. We show that endogenous DDIT4 expression is upregulated in clonally derived MSCs with high differentiation potential and are in turn associated with reduced mTOR signalling when compared to MSC populations with endogenously low *DDIT4* expression levels. Furthermore we show that DDIT4 is activated downstream of *HIF1α* in response to hypoxia and p53 pathways. In addition, we demonstrate that DDIT4 activity is directly linked to regulation of mTOR signalling, expression of pluripotency genes, differentiation and proliferation of MSCs and mesenchymal progenitor cells.

## Results

### Gene expression of *DDIT4* is associated with MSCs with high differentiation potentials

**Figure 1:**
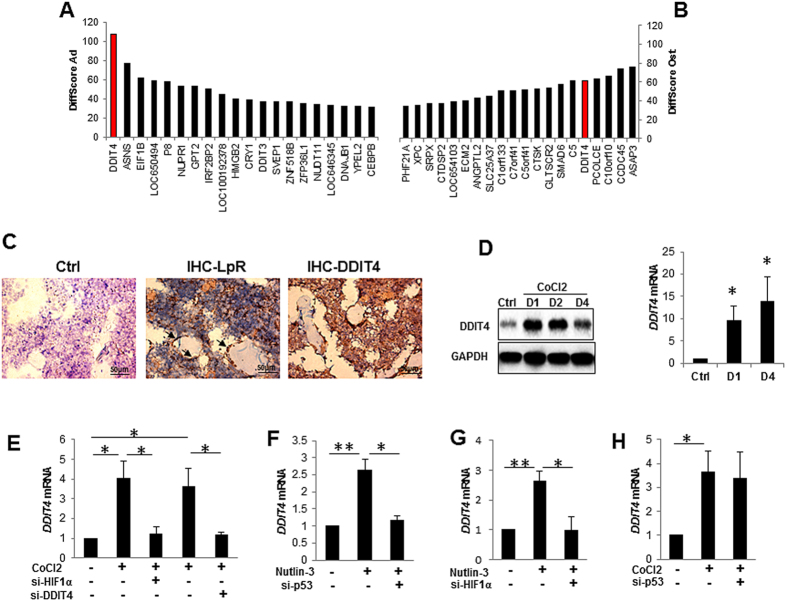
Expression of *DDIT4* gene regulates MSC function downstream of *HIF1α*. (**A**,**B**) Global gene expression analysis of MSC derived from single clones with different differentiation potentials showing the top 20 genes highly expressed in clones with high capacity for (**A**) adipogenic and (**B**) osteogenic differentiation. The data were processed for the background signal, normalized, and differences in gene expression were compared by the Differential Expression Algorithm using the Illumina custom error model of GenomeStudio software (Illumina, Inc.). Genes with a Diffscore of ±13, equivalent to *p* < 0.05, were considered as being significantly up-regulated; a DiffScore of ±20, ±30 or ±40 was equivalent to a *p*-value of <0.01, <0.001, or <0.0001, respectively. (**C**) Immunohistochemistry staining for Leptin Receptor (LpR) and DDIT4 proteins in the bone marrow of wild type mice showing (left) sham control, (middle) LpR or (right) DDIT4 staining of bone marrow sections. (**D**,**E**) Expression of DDIT4 in MSC and in response to hypoxia mimetic (CoCl2). (**D**) MSC were treated with CoCl2 for 1 day (D1) or 4 days (D4) and relative expression of DDIT4 was determined by western blotting (left panel) or qRT-PCR (right panel) (n = 5). (**E**–**H**) Expression of *DDIT4* in MSC treated with CoCl2 (100 μM) or Nutlin3 (20 μM) after siRNA knock-down of *HIF1α*, *p53* or *DDIT4* or transfection with scrambled control as negative control. MSC were transfected with *HIF1α* or *DDIT4* or control siRNA for 24 h and subsequently treated for a further 24 h with CoCl2 or Nutlin3 or vehicle control prior to qRT-PCR analysis for *DDIT4* (n = 4). Error bars indicate mean ± SEM. *p < 0.05; **p < 0.01.

MSC are a heterogeneous cell population with wide variations in behaviour^18^,^19^. The heterogeneity of stem cell populations is linked to cell intrinsic differences that determine the responses of the cells to environmental cues which affect self-renewal, differentiation, quiescence and aging^20^. To investigate the intrinsic mechanisms involved in MSC self-renewal and multipotency, we derived clonal MSC cultures by limiting dilution and characterised their differentiation potentials as having high osteogenic potential, high adipogenic potential or low differentiation potentials. (Fig. S1A,B). Global gene expression analysis showed respectively 201 and 339 differentially regulated genes in adipogenic and osteogenic clones compared to clones with low differentiation capacities. Amongst these differentially expressed genes, *DDIT4* was observed as the first gene of 100 and the fifth gene of 124 genes whose expression was consistently higher in clonal populations with strong differentiation capacity to adipocyte and osteoblast lineages respectively (Figs 1A,B and S1E,F). QRT-PCR analysis validated these and also showed that the same is true for the clones with multi-differentiation potential (Fig. S1G,H). In order to demonstrate DDIT4 expression *in situ* we co-localised DDIT4 expression to MSC populations within the bone marrow by immunohistochemistry using Leptin Receptor (LepR) expression as a marker for the identification of MSCs^21^. Sections from bone marrow of wild-type mice exhibited strong staining for both LepR and DDIT4 (Fig. 1C). LepR and DDIT4 staining were distributed throughout the bone marrow. However DDIT4 staining was more widespread than LepR, possibly suggesting that other cells types may also express DDIT4 within the hypoxic bone marrow environment.

### DDIT4 expression acts downstream of *HIF1α*

We used CoCl2 to mimic a hypoxic environment in MSC cultures. The level of both DDIT4 mRNA and protein were significantly up-regulated following treatment with CoCl2 (Fig. 1D). Next we determined whether DDIT4 acts as a downstream regulator of the main regulator of hypoxic responses, HIF1α. siRNA knock-down of either *HIF1α* or *DDIT4* completely reversed the stimulatory effect of CoCl2 to basal levels (Fig. 1E). p53 is also implicated in the regulation of DDIT4 in a number of cell lines^22^. We found that activation of the p53 pathway by p53 stabilization using Nutlin-3 significantly up-regulated the expression of DDIT4 in MSC. This stimulatory effect was completely neutralised when *p53* was knocked down (Fig. 1F). Interestingly, loss of *HIF1α* also reversed the effect of p53 activation on MSC (Fig. 1G). In contrast, p53 knock-down had no effect on CoCl2-induced DDIT4 up-regulation, suggesting that HIF1α regulates DDIT4 downstream of both hypoxia and p53 (Fig. 1H).

### DDIT4 regulates mTOR signalling and MSC stemness properties

**Figure 2:**
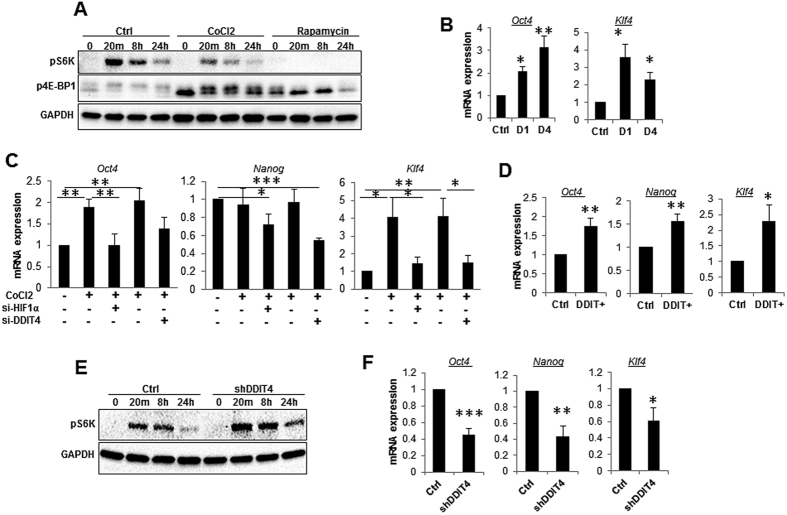
DDIT4 regulates mTOR singling and the expression of pulripotency genes in MSC. (**A**) MSC were pre-treated with CoCl2 (100 μM), rapamycin (10 nM) or vehicle control for 24 hours, serum starved for 6 hours and phosphorylation of S6K, and 4E-BP1were analyzed in response to stimulation by media containing 10% serum for 20 minutes, 6 and 24 hours using Western blotting. CoCl2 and rapamycin were present at all time during serum deprivation and stimulation period. Data shown is representative of three separate experiments. (**B**–**F**) Expression of pluripotency genes *Oct4*, *Nanog* and *Klf4.* (**B**) MSC were treated with 100 μM of CoCl2 for 1 day (D1) or 4 days (D4) and expression of *Oct4* and *Klf4* was determined by qRT-PCR (*n* = 5). (**C**) MSC were transfected with *HIF1α* or *DDIT4* or control siRNA for 24 h and subsequently treated for a further 24 h with CoCl2 (100 μM) prior to qRT-PCR analysis (*n* = 5). (**D**) DDIT4 was transiently overexpressed in MSC and the expression of *Oct4*, *Nanog* and *Klf4* was determined by qRT-PCR (*n* = 5). (**E**) MSC stably transfect with shRNA against *DDIT4* or negative control were analysed for mTOR substrate (p-S6K) activation and (**F**) the expression of pluripotency genes *Oct4*, *Nanog* and *Klf4*. Error bars indicate mean ± SEM. *p < 0.05; **p < 0.01; ***p < 0.001.

Given the importance of DDIT4 in the regulation of the mTOR pathway, we next examined the effect of CoCl2-induced DDIT4 expression on activation of mTOR. As shown in Fig. 2A, CoCl2 treatments led to reductions in phosphorylation of the mTOR substrate pS6K and hypo-phosphorylation of 4E-BP1. This is a similar, but less potent, effect to that seen with rapamycin (Fig. 2A).

We have previously shown that inhibition of mTOR significantly increases the expression of pluripotency genes *Oct4* and *Nanog* and prevent loss of self-renewal and function of MSC during aging^12^. It has been also demonstrated that a hypoxic environment maintains self-renewal and stemness properties of MSC by inducing the expression of *Oct4*, *Nanog* and *Klf4*^23^. In keeping with these observations and with DDIT4 being a regulator of mTOR signalling in response to hypoxia, we found that DDIT4 acts downstream of HIF1α and hypoxia to regulate these pluripotency genes. As with *DDIT4* expression, levels of *Oct4* and *Klf4* were strongly up-regulated when hypoxic signalling was initiated by the presence of CoCl2 (Fig. 2B). However, knock-down of either *DDIT4* or *HIF1α* prior to treatment with CoCl2 completely blocked the induction of these pluripotency genes and their expression remained at basal levels (Fig. 2C). The expression of *Nanog* was also reduced by *DDIT4* or *HIF1α* knock-down, despite being unchanged following CoCl2 treatment (Figs 2C and S2F).

To further confirm that DDIT4 is responsible for the observed effect of hypoxia on MSC, we transiently overexpressed the *DDIT4* in MSCs in normoxic conditions and this resulted in significant up-regulation of *Oct4*, *Nanog* and *Klf4* genes (Fig. 2D). Conversely, stable shRNA knock-down of *DDIT4* resulted in a significant reduction in the expression of these genes even under normoxic conditions, suggesting that DDIT4 is at least partially constitutively active even in the absence of a stimulus in MSCs (Fig. 2F). This was further supported by the observation that *DDIT4* shRNA knock-down was accompanied by elevated mTOR signalling as shown by reduced S6K dephosphorylation, particularly at late time points (Fig. 2E). Collectively our data demonstrate that HIF1α or mTOR dependent expression of pluripotency genes in MSC is regulated by DDIT4, both under the basal condition or following stimulation.

### DDIT4 inhibits MSC differentiation

**Figure 3:**
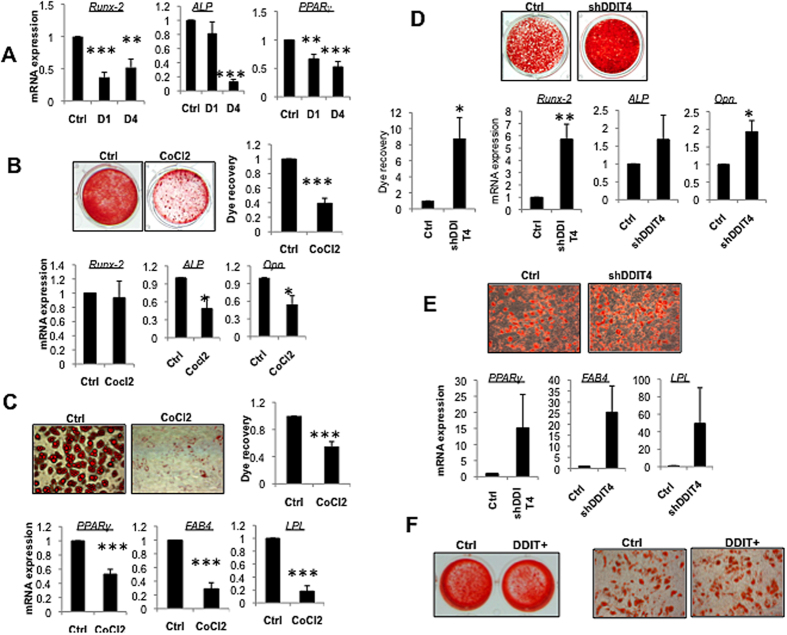
DDIT4 regulate MSC differentiation. (**A**) Expression of vital genes involve in osteogenic and adipogenic differentiation in MSC treated with CoCl2 or vehicle control (*n* = 4). (**B**) Osteogenic differentiation of MSC in presence of CoCl2. (Upper left) matrix mineralization visualized and (upper right) quantified following Alizarin Red staining (representative images from four independent experiments) and (lower panel) mRNA expression of the osteogenic markers *Runx2*, *ALP*, and *Osteopontin (OPN*) was analyzed by qRT-PCR (*n* = 5). (**C**) Adipogenic differentiation of MSC in presence of CoCl2. (Upper left) Lipid accumulation was visualized and (upper right) quantified following Oil red O staining (×200) and (lower panel) expression of adipogenic markers *PPARγ*, *FAB4*, and *LPL* mRNA was analyzed by qRT-PCR (*n* = 5). (**D**) Osteogenic differentiation of MSC in *DDIT4* depleted cells. MSC stably transfect with shRNA against *DDIT4* or negative control were differentiated to osteoblast and (upper) matrix mineralization was visualized and (lower left) quantified by Alizarin Red staining and (lower right) mRNA expression of the osteogenic markers was analyzed by qRT-PCR (*n* = 4). (**E**) Adipogenic differentiation of MSC in *DDIT4* depleted cells. MSC stably transfect with shRNA against *DDIT4* or negative control were induced to differentiate to adipocytes and the ability of (upper left) lipid accumulation was visualized (×200) and (lower panel) expression of adipogenic markers was analyzed by qRT-PCR (*n* = 4). (**F**) Differentiation following transient overexpression of DDIT4. (Left) Alizarin Red staining of matrix mineralization and (right) Oil Red O staining for lipid droplets following differentiation to osteoblast and adipocyte lineages (×200). Error bars indicate mean ± SEM. *p < 0.05; **p < 0.01; ***p < 0.001.

Excessive activation of mTOR signalling is suggested to drive unwanted differentiation and proliferation. mTOR activation has been shown to accelerate differentiation of MSCs and may result in depletion of the stem cell pool. Therefore we next examined the differentiation capacity of MSCs following manipulation of DDIT4 by either CoCl2 activation and gene knock-down or overexpression. Treatment with CoCl2 significantly reduced the expression of genes associated with osteoblast (*Runx2* and *ALP*) and adipocyte (*PPARγ*) lineages in the absence of any differentiation stimulus (Fig. 3A), suggesting a reduction in spontaneous differentiation by MSC. Similarly when MSC were induced to differentiate in the presence of CoCl2, they showed a significant reduction in differentiation (Fig. 3B,C). Expression of markers of osteogenic (Fig. 3B) and adipogenic (Fig. 3C) differentiation were significantly reduced and the ability of the cells to undergo matrix mineralization (Fig. 3B) or the transformation to lipid containing adipocytes (Fig. 3C) was strongly impaired. We also stably knocked down *DDIT4* which resulted in accelerated MSC differentiation toward both lineages, as determined by elevated expression of genes associated with osteoblasts (Fig. 3D) and adipocytes (Fig. 3E), increased matrix mineralization (Fig. 3D) and formation of lipid containing adipocytes (Fig. 3E) in response to stimulation of differentiation. However, no significant effect was observed under basal conditions (Fig. S2G). Interestingly, transient overexpression of DDIT4 in MSC had only a negligible effect on differentiation, which may be due to the temporary nature of expression (Fig. 3F).

**Figure 4:**
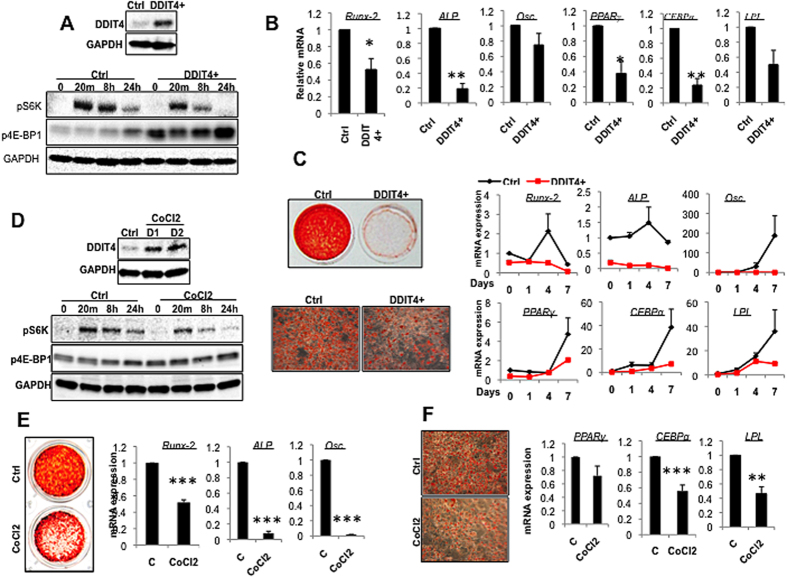
DDIT4 expression and activation inhibits the differentiation of progenitors. (**A**) (Top panel) Expression of DDIT4 protein and (lower panel) phosphorylation of mTOR substrate S6K, and 4E-BP1 in control and DDIT4 overexpressing 7F2 progenitor cells. (**B**) Expression of genes involved in osteogenic and adipogenic lineage commitment in undifferentiated control and DDIT4 overexpressing 7F2 cells. (**C**) Differentiation capacity of 7F2 overexpressing DDIT4 or control plasmid constructs. (Upper right) mineralization visualized following Alizarin Red staining and (upper left) mRNA expression of the osteogenic markers (*Runx2*, *ALP*, and *Osteocalcin (Osc*)) analyzed by qRT-PCR (*n* = 3). (Lower right) Oil red O staining for lipid droplets in adipocytes (×200) and (lower right) expression of adipogenic markers (*PPARγ*, CEBPα and *FAB4*) analyzed by qRT-PCR (*n* = 3). (**D**) (Top panel) Expression of DDIT4 and (lower panel) phosphorylation of mTOR substrate in progenitor cells treated with hypoxia mimic. 7F2 cells were treated with 100 μM of CoCl2 for 1 and 2 days and expression of DDIT4 was determined by western blotting (top panel). For pathway analysis cell were pre-treated with CoCl2 for 24 hours and phosphorylation of S6K, and 4E-BP1were analyzed in response to stimulation with serum in presence of CoCl2. (**E**,**F**) Differentiation capacity of 7F2 cell treated with hypoxia mimic CoCl2. (**E**) Osteogenic differentiation was assessed by Alizarin Red staining of matrix mineralization and mRNA expression of the osteogenic associated genes by qRT-PCR (*n* = 3). (**F**) Adipogenic differentiation was assessed by Oil red O staining for lipid droplets in adipocytes (×200) and by expression of adipogenic markers (*n* = 3). Error bars indicate mean ± SEM. *p < 0.05; **p < 0.01; ***p < 0.001.

p53 signalling has been shown to be vital regulator of MSC proliferation and differentiation and has also shown here to be implicated in regulation of *DDIT4*. To further explore the role of DDIT4 in quiescence, self-renewal and mesenchymal linage differentiation, we used a bone marrow mesenchymal stem/progenitor cell line (7F2) derived from *p53*^*−/−*^ mice. The cell line is spontaneously immortalized and shows markedly increased differentiation to both osteoblast and adipocyte lineages^24^. As with any immortalised cell line the phenotypic characteristics of 7F2 cells may not completely reflect the features of primary MSC in term of proliferation rate and differentiation potential. 7F2 cells showed extremely low levels of DDIT4 expression and high levels of lineage specific marker expression and greatly elevated differentiation capacity (Fig. 4). In line with the low DDIT4 level, further depletion of *DDIT4* gene by siRNA knock-down had no effect on the function of 7F2 cells (Fig. S3A).

To determine whether DDIT4 is able to regulate differentiation in committed progenitors, we stably overexpressed DDIT4 in these progenitor cells. As shown in Figs 4A and S3B, overexpression of DDIT4 was associated with a marked reduction in mTOR activity as measured by phosphorylation of mTOR substrate. DDIT4 overexpression in basal conditions also led to a significant reduction in expression of genes associated with differentiation phenotype, notably transcription factors involved in the early stages of lineage commitment (*Runx2*, *PPARγ* and *CEBPα*) (Fig. 4B) and greatly impaired the ability of the cells to differentiate to either osteoblast and adipocyte lineages (Fig. 4C). Cells overexpressing DDIT4 showed a significant loss of capacity for matrix mineralization and marked reduction in lipid accumulation, accompanied by reduction or complete loss of differentiation-induced expression of genes involved in osteogenesis or adipogenesis (*Runx2*, *ALP*, *osteocalcin (Osc*), *PPARγ*, *CEBPα* and *LPL*) (Fig. 4C). In support of these observations, DDIT4 protein was strongly induced in 7F2 cells in response to CoCl2 and this up-regulation correlated with the suppression of mTOR activity (Fig. 4D). CoCl2 induced DDIT4 expression led to marked reduction in capacity of 7F2 cells to undergo differentiation to either osteoblast (Fig. 4E) and adipocyte (Fig. 4F) lineages. Taken together these results indicate that DDIT4 plays a vital role in maintenance of the undifferentiated state and stemness properties of MSC and 7F2 mesenchymal progenitors by suppressing spontaneous or excessive differentiation.

### Endogenous *DDIT4* level maintains MSC function

**Figure 5:**
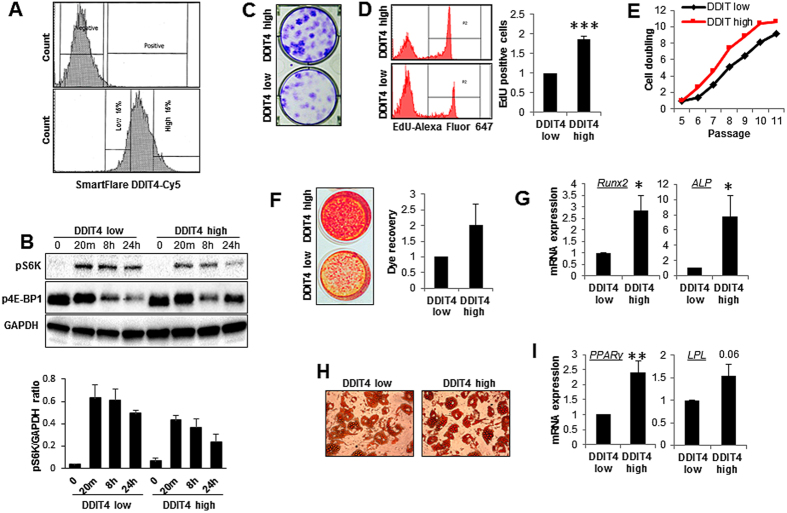
Endogenous DDIT4 level governs MSC fate and function. SmartFlare™ live cell DDIT4 RNA sorting and functional analysis of DDIT4-low and DDIT4-high sub-populations. (**A**) Isolation of MSC subsets based on *DDIT4* expression using SmartFlare RNA detection probes and fluorescence activated cell sorting. MSC were isolated into DDIT4-low and high populations by sorting the lowest and highest 15% of the population based on APC fluorescence. (**B**) MSC were serum starved for 6 hours and phosphorylation of S6K, and 4E-BP1 were analyzed in response to stimulation by media containing 10% serum for 20 minutes, 6 and 24 hours using Western blotting. Data shown is representative of four separate experiments. (lower panel) Quantitative densitometry of pS6K normalised to GAPDH. (**C**) The frequency of colony formation by each population was assessed by colony forming unit assay and crystal violet staining (representative images from five independent experiments). (**D**) Differences in rate of cell cycle were determined by Click-iT EdU assay following 48 hours of incubation using flow cytometry (*n* = 4). (**E**) Cumulative cell doubling per passage was calculated by cell counting (*n* = 5). (**F**) Osteogenic differentiation capacity was determined by Alizarin Red staining of mineralized matrix and (**I**) mRNA expression of the osteogenic markers *Runx2* and *ALP* was assessed by qRT-PCR (*n* = 5). (**J**) Adipogenic differentiation was analyzed following Oil Red O staining for lipid droplets (×200) and (**K**) expression of adipogenic markers *PPARγ* and *LPL* by qRT-PCR (*n* = 5). Error bars indicate mean ± SEM. *p < 0.05; **p < 0.01; ***p < 0.001.

MSCs were sorted into two subpopulations with high and low *DDIT4* mRNA expression levels using SmartFlare mRNA probes and fluorescence activated cell sorting (FACS) (Fig. 5A). The DDIT4-low and -high populations displayed contrasting levels of mTOR signalling following stimulation (Fig. 5B), as measured by levels of phosphorylation of substrates. In particular S6K phosphorylation was considerably higher in DDIT4-low compared with DDIT4-high cells. In line with studies that have reported an inverse association between mTOR activity and self-renewal and longevity, the DDIT4-high population showed greater stem cell properties with high clonogenic capacity (Fig. 5C), proliferation rate (Fig. 5D) and increased osteogenic and adipogenic differentiation following transfer of cells to differentiation media (Fig. 5F–I). The *DDIT4*-high population showed increased expression of markers of differentiation for both osteoblast (*Runx2* and *ALP*) (Fig. 5G) and adipocyte (*PPARγ* and *LPL*) (Fig. 5I) lineages and produced higher matrix mineralization (Fig. 5F) and lipid containing adipocytes (Fig. 5H) when cells were induced to differentiate. These observations are in agreement with data obtained with the single clone analysis reported at Fig. 1A,B. The DDIT-high population also showed a greater number of cell doublings with long-term continuous culture (Fig. 5E). The data presented here are in line with previous studies in which long term inhibition of mTOR and the excessive differentiation prevent aging and maintained stemness of number of stem cell types^6^,^9^,^10^,^11^,^12^.

## Discussion

Adult stem cells reside in niches within the tissues where they are kept quiescent and slow cycling to maintain stem cell characteristics within the lifespan of an organism. Stem cell fate decisions to remain quiescent, self-renew or differentiate are largely governed by extracellular signals from the niche that manipulate cell intrinsic signal cascades and transcriptional programs^25^. Hypoxia (low oxygen tension) is widely proposed as a critical regulatory component of the niche for several adult stem cell pools including mesenchymal and hematopoietic stem cells^26^. MSC are localised to relatively hypoxic perivascular microenvironments *in vivo* and similar levels of oxygen tension preserve their stemness *in vitro*^21^,^27^,^28^. The responses to hypoxia are mainly due to the regulation of HIF1α induced expression of pluripotency genes *Oct4* and *Nanog* and reduced intracellular reactive oxygen species^23^. Suppression of the mTOR pathway also maintains the quiescence and function of MSC, HSC and epithelial stem cells by reducing the reactive oxygen species and oxidative stress^10^,^11^,^12^. Activity of the mTOR pathway has been shown to be higher in HSC from aged mice and increasing mTOR signalling is sufficient to promote premature aging of HSC in young mice. Together these observations suggest that HIF1α and mTOR signalling are important cell intrinsic component of the niche for stem cells^8^.

In this study we show a possible role for DDIT4 in regulation of MSC function in response toHIF1α and mTOR pathway. Using whole genome microarray analysis, gene expression profiles of cells derived from single clones with distinct differentiation potentials demonstrated that *DDIT4* expression was markedly higher in clonal populations that retained a higher differentiation capacity. High level of DDIT4 in both uni-potential and multi-potential clones and the widespread staining of DDIT4 within the cells in the bone marrow suggest the involvement of DDIT4 in committed progenitor cells. DDIT4 can inhibit mTOR in response to both hypoxia and nutrient restriction^16^,^17^. In the absence of DDIT4, phosphorylation of TSC by Akt initiates the binding of 1433 and prevents the inhibition of mTOR by TSC1/2. DDIT4 competes with TSC2 for 1433 and stabilizes the complex formed by TSC1/2, thereby inducing the inhibition of mTOR^16^. We demonstrate that DDIT4 expression and activation negatively regulate mTOR and regulates MSCs functions. Induction of the *DDIT4* gene by the hypoxia mimetic CoCl2 significantly down-regulated the mTOR signal and resulted in lower levels of differentiation, while *DDIT4* knock-down was associated with higher differentiation rate. These findings may indicate that high DDIT4 activity is inhibitory to excess differentiation and lead to maintenance of stem cell function Our data also suggest a possible link between endogenous levels of DDIT4 with maintenance of differentiation capacity of MSC. Sorted MSC subpopulations with high endogenous DDIT4 expression also exhibited enhanced clonogenic capacity, self-renewal rate and differentiation potentialThese findings is in line with the observation of significant exhaustion of the HSC population located in a more oxygenic vascular niche with high mTOR expression, when compared to more self-renewing populations with lower mTOR activity^29^.

In addition to the effect on differentiation, we showed that DDIT4 also positively regulates the expression of pluripotency genes. These effects have been previously observed to be regulated by HIF1α in response to low oxygen tension^23^. HIF1α is widely known as the master regulator of cellular response to hypoxia through regulation of transcriptional activities^30^. In MSC, hypoxia induced HIF1α action was mediated through activated *Twist* with direct down-regulation of *E2A-p21* and subsequent induction of transcription factors such as *Oct4* and *Nanog* that control stem cell self-renewal and multipotency^23^. Although the transcriptional aspect of HIF1α induced regulation of pluripotency is well described, little is known about the signal pathways involved. Here we show that these effects of HIFα, at least in MSCs, are mediated byDDIT4 activation and subsequent mTOR inhibition. Loss of *HIF1α* completely abolished the hypoxia mimic-induced expression of *DDIT4* expression and the knock-down of either *HIF1α* or *DDIT4* inhibited the induced expression of pluripotency genes. It is interesting that inhibition of mTOR by rapamycin also induces *Oct4* and *Nanog* expression, while activation of mTOR by either *Tsc2* depletion or *Tsc2* knock-down greatly reduce the reprogramming of somatic cells to induced pluripotent stem (iPS) cells and mTOR activity has been shown to be higher in fibroblasts than in embryonic stem cells and iPSCs^12^,^31^.

In addition to the connection with HIF1α, we observed that DDIT4 also acts downstream of the p53 pathway. Activation of the p53 pathway was associated with significant up-regulation of *DDIT4* expression, which was abolished when *p53* was knocked down. It is well documented that loss of *p53* is associated with spontaneous and accelerated differentiation in MSCs. MSCs or progenitor cells lacking *p53* show high levels of differentiation to both osteoblast and adipocyte lineages^32^,^33^. These phenotypical behaviours may also be seen with other immortalised cell lines. Nonetheless, our results indicate that progenitors lacking *p53* show a very low level of DDIT4 expression. Induction of DDIT4 by either chemically induced hypoxia or overexpression significantly suppressed the differentiation capacity of these committed progenitor cells. As mentioned above the immortalised progenitors are distinct to wt murine MSCs in respect of differentiation and proliferation capacities and this should be taken in consideration.

In summary, stem cells are dependent on their ability to maintain their pool whilst also being able to respond to physiological and pathological conditions to repair and renew tissues throughout the lifetime of the organism. Thus stem cells require tightly regulated intrinsic mechanisms to coordinate between states of quiescence, self-renewal and differentiation. Here, we show that DDIT4 is a critical regulator of mTOR signalling and MSC functionsin response to HIF1α and the upstream signals Our study is however limited to *in vitro* experimentation and chemically induced hypoxic culture conditions. Future studies with controlled hypoxic culture conditions with various oxygen gradients as well as *in vivo* studies of the effect of DDIT4 knock out on maintenance of MSC in aging is needed to confirm and further extend our findings.

## Materials and Methods

### Cell culture

Primary human MSCs were purchased from Lonza and 7F2 progenitor cell line (ATCC) was a kind gift from Dr. Bronwen Evans. Detailed cell culture methods, single-clone culture, lineage differentiation, cell proliferation and viability assay, and immunostaining are provided in SI experimental procedures.

### Microarray analysis

Global gene expression analysis was performed using the Illumina whole-genome expression array HumanHT-12 v4.0 Expression BeadChip according to the manufacturer’s instructions. The Beadchips were scanned on the Illumina iScan System with iScan software. The raw data were processed for the background signal, normalized, and differences in gene expression were compared by the Differential Expression Algorithm using the Illumina custom error model of GenomeStudio software (Illumina, Inc.). For details see SI experimental procedure.

### Live cell RNA sorting

Cell were sorted based on DDIT4 RNA expression level using SmartFlare™ RNA detection probes (SmartFlares; Millipore) and a FACSAria cell sorter (BD Bioscience) into two population. Sorted sub-populations were then returned to cell culture and expanded for further analysis. For details see SI experimental procedure.

### Transfection

shRNA Plasmids and siRNAs were purchased from (Qiagen) and full-length DDIT4 construct was purchased from (Life Technologies) and used according to according to manufacturer’s instruction. Transfection was performed using the Neon transfection system (Life Technologies) according to the manufacturer’s instructions. For details see SI experimental procedure.

### Immunoblotting

Cells were lysed in RIPA lysis buffer, protein lysates were separated by SDS–PAGE, transferred onto PVDF membranes, subjected to immunoblotting and detected by ECL. For details see SI experimental procedure.

### Quantitative Reverse Transcription Polymerase Chain Reaction (qRT-PCR)

RNA was isolated using TRI reagent (Life Technologies) and cDNA was prepared using QuantiTect Reverse Transcription Kit (Qiagen). qRT-PCR was performed on a Mx3000P real time PCR system using SYBR Green qPCR Master mix (BioRad). Detailed methodology and primers information are provided in SI experimental procedure.

## Additional Information

**How to cite this article**: Gharibi, B. *et al.* DDIT4 regulates mesenchymal stem cell fate by mediating between HIF1α and mTOR signalling. *Sci. Rep.* **6**, 36889; doi: 10.1038/srep36889 (2016).

**Publisher’s note:** Springer Nature remains neutral with regard to jurisdictional claims in published maps and institutional affiliations.

## Supplementary Material

Supplementary Information
