# Supplementary material for: DDIT4 regulates mesenchymal stem cell fate by mediating between HIF1α and mTOR signalling
Source: Sci Rep. 2016 Nov 23;6:36889. doi: 10.1038/srep36889 (PMC5120275; doi:10.1038/srep36889)
Supplement: Supplementary Information [file srep36889-s1.pdf]

Title

DDIT4 regulates mesenchymal stem cell fate by mediating the interaction between HIF1α and mTOR signalling.

Authors/Affiliations

Borzo Gharibi, Mandeep Ghuman and *Francis J Hughes

Division of Tissue Engineering and Biophotonics, Dental Institute, King's College London, Tower Wing, Guy’s Hospital, London, SE1 9RT. UK

S1

A

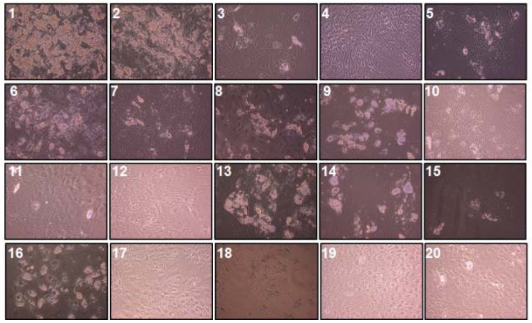

B

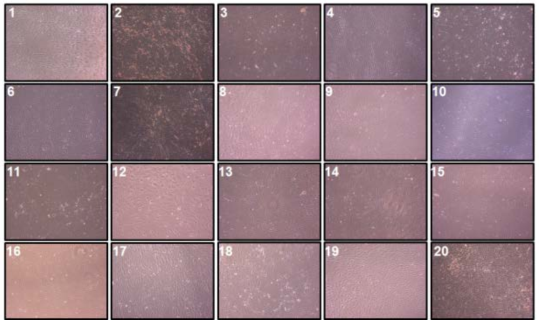

C

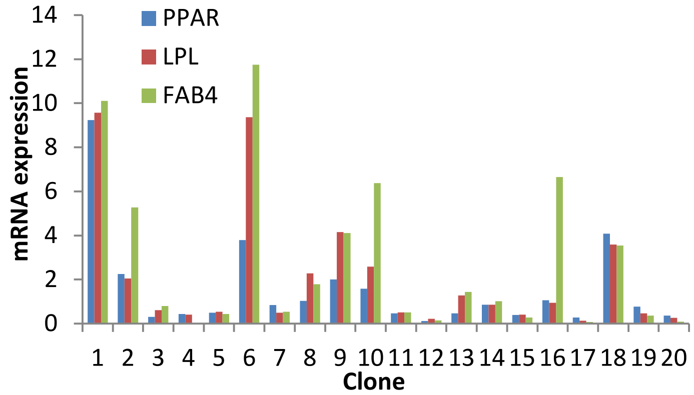

D

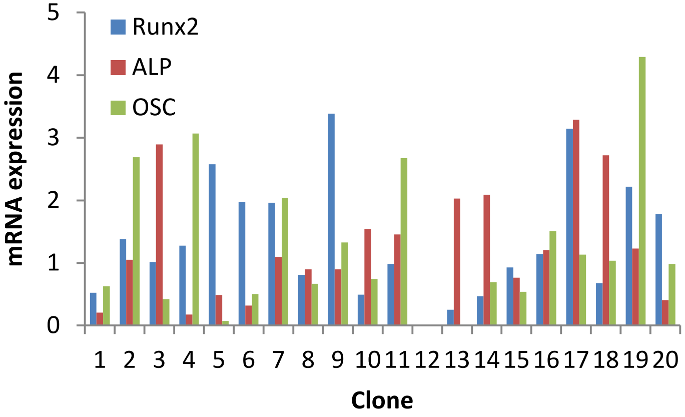

G

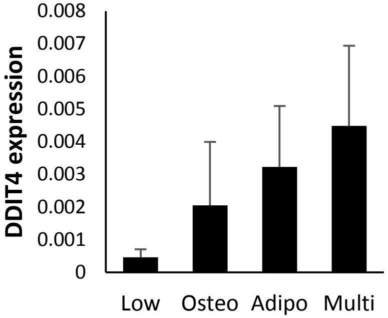

H

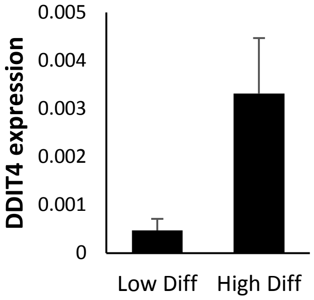

E

	SYMBOL		SYMBOL
1	DDIT4	51	ASAP3
2	ASNS	52	EXOSC8
3	EIF1B	53	NPIP
4	LOC650494	54	LOC644935
5	P8	55	RSL24D1
6	NUPR1	56	DVL2
7	GPT2	57	KLHDC5
8	IRF2BP2	58	DHRS3
9	LOC100192378	59	EMX2OS
10	HMGB2	60	ADCY3
11	CRY1	61	FLJ10081
12	DDIT3	62	RYBP
13	SVEP1	63	CCDC45
14	ZNF518B	64	ZFPM1
15	ZFP36L1	65	C14orf167
16	NUDT11	66	CLK4
17	LOC646345	67	CRBN
18	DNAJB1	68	LOC286157
19	YPEL2	69	EAPP
20	CEBPB	70	PEX11G
21	SERTAD4	71	C2orf64
22	GPX4	72	PTEN
23	PDGFRB	73	AUTS2
24	ATF4	74	LOC100134424
25	DACT3	75	DPYSL2
26	LONRF1	76	PHF21A
27	IER5L	77	LOC400657
28	CMTM8	78	SETBP1
29	CEBPG	79	NCRNA00219
30	CARHSP1	80	BTF3L4
31	GPR124	81	C1RL
32	LACTB2	82	PRICKLE1
33	TIGA1	83	RFX7
34	RNF217	84	HSF2
35	IGF2BP2	85	VPS37D
36	SIN3A	86	MEIS1
37	PCMTD1	87	PPM1D
38	THBS3	88	C9orf91
39	BRD3	89	RHOD
40	KIAA0831	90	SFRS8
41	ZCCHC24	91	TRIM25
42	ARHGEF6	92	CTSF
43	VGLL4	93	CCDC102A
44	WDR48	94	SHMT2
45	FCGRT	95	ANKRA2
46	BTG1	96	NID1
47	IRS2	97	LOC730820
48	SESN2	98	TRIB3
49	CCDC109A	99	PCDH18
50	CYP2R1	100	MARS

F

	SYMBOL		SYMBOL
1	ASAP3	62	PIGC
2	CCDC45	63	NUDT11
3	C10orf10	64	EXOSC8
4	PCOLCE	65	ZC3H5
5	DDIT4	66	KCTD3
6	C5	67	LOC644914
7	SMAD6	68	C14orf167
8	GLTSCR2	69	CNTNAP1
9	CTSK	70	PRKRIR
10	C5orf41	71	KANK2
11	C7orf41	72	SSBP2
12	C1orf133	73	MRPS6
13	SLC25A37	74	LOC100134424
14	ANGPTL2	75	TULP3
15	ECM2	76	CBARA1
16	LOC654103	77	SETBP1
17	CTDSP2	78	ARL2
18	SRPX	79	ABHD8
19	XPC	80	FLJ46906
20	PHF21A	81	TSPAN9
21	CCDC102A	82	LOC400027
22	KIAA0831	83	IRX3
23	EIF1B	84	PTEN
24	SERTAD4	85	VPS37D
25	COL14A1	86	PHF13
26	P8	87	TBL1XR1
27	ANKRD10	88	COL16A1
28	PLEKHF1	89	CBS
29	GPR124	90	LOC653778
30	OLFML3	91	AUTS2
31	DVL2	92	LOC727882
32	GUSB	93	ZNF395
33	RBM17	94	GBP2
34	PDGFRA	95	KAZALD1
35	ZCCHC24	96	LOC728153
36	SAP30	97	OFD1
37	THBS3	98	ZSWIM4
38	FCGRT	99	SIN3A
39	HMGB2	100	LOC100128892
40	PLSCR4	101	SIX5
41	EIF4B	102	SLC37A4
42	EVL	103	AEBP1
43	PCDH18	104	CCDC109B
44	BTF3L4	105	BTG1
45	PIGT	106	JDP2
46	WDSUB1	107	TMEM119
47	KIAA0146	108	KIAA0528
48	SLC44A1	109	LRCH3
49	NUPR1	110	PCMTD2
50	CCDC80	111	GPX4
51	CRBN	112	H2AFY2
52	FAM164A	113	WDR19
53	TRIM22	114	C1RL
54	SAV1	115	DNAJC27
55	PFAAP5	116	RSL24D1
56	C1S	117	ZFP90
57	ZFP161	118	LOC647673
58	SLC35A1	119	HRASLS3
59	RGL1	120	CCDC106
60	H2AFZ	121	PROS1
61	IGF2BP2	122	PRICKLE1

S1. Global gene expression analysis of MSC derived from single clone with different differentiation potency. (A-D) MSC single clones were stablished by limiting dilution of the cell suspension. The multi-potential capacity to (A) adipogenic and (B) osteogenic linages was determined by morphological examination and (C&D) gene expression analysis of the adipogenic (PPARγ, LPL and FAB4) and osteogenic (Runx2, ALP and Osteocalcin) markers by qRT-PCR. Clones considered having high osteogenic (4, 7, 19), high adipogenic (1, 8, 18) or low differentiation potentials (12, 14, 15). (E&F) Global gene expression was carried out using the Illumina microarray platform and differences in gene expression were compared by the Differential Expression Algorithm using the Illumina custom error model of GenomeStudio software (Illumina, Inc.). Genes with a Diffscore of ±13, equivalent to $p<0.05$, were considered as being significantly upregulated; a DiffScore of ±20, ±30 or ±40 was equivalent to a p-value of <0.01, <0.001, or <0.0001, respectively. Cluster of genes highly expressed in (E) osteogenic and (F) adipogenic clones compare with clones with low differentiation capacity. (G) QRT-PCR analysis of DDIT4 expression in clones with high osteogenic (4, 7, 19), high adipogenic (1, 8, 18), high multi (2, 6, 9) or low differentiation potentials (12, 14, 15). (H) Combined data showing DDIT4 expression in clones with high or low differentiation potentials.

S2

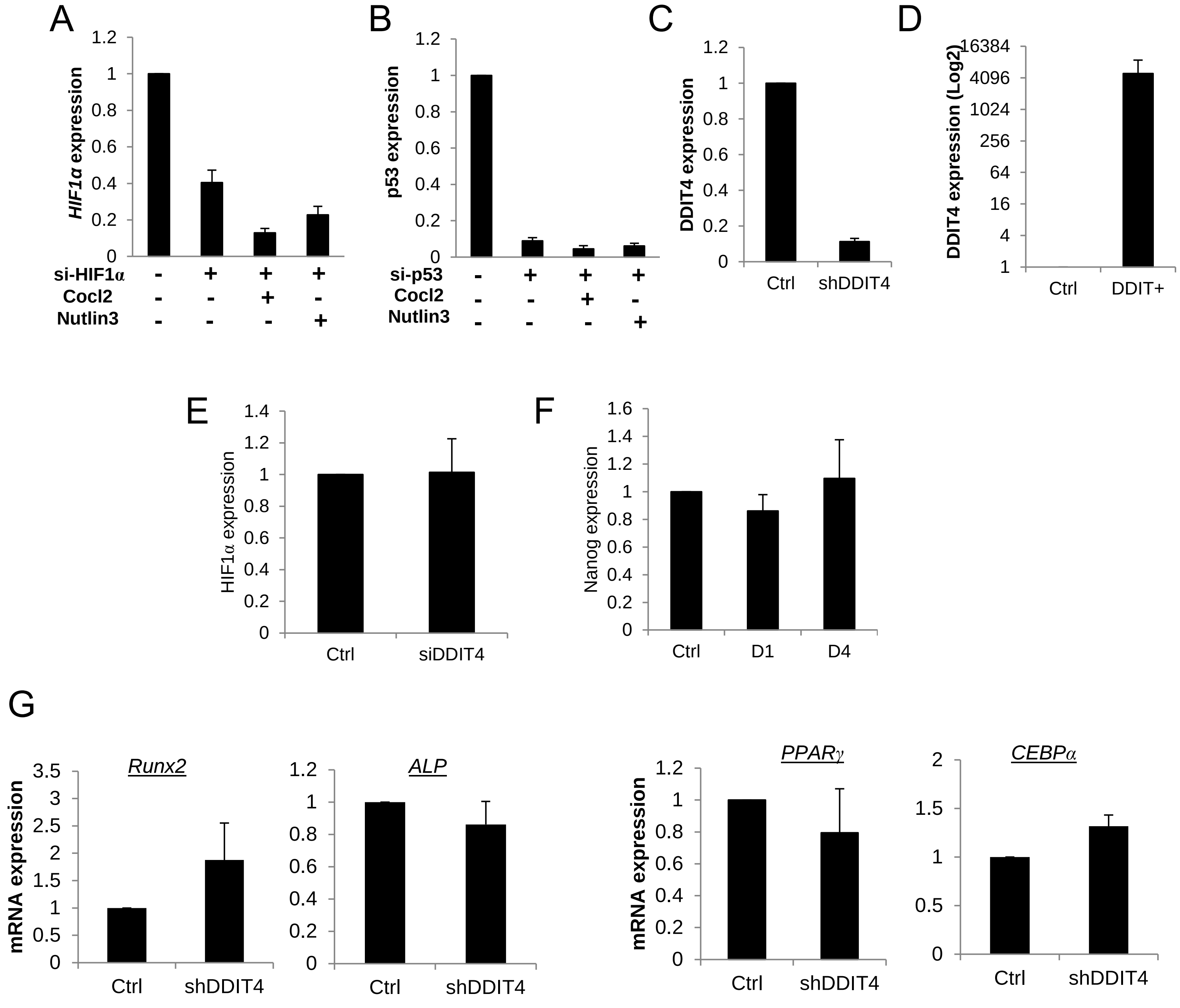

A
HIF1α expression
si-HIF1α - + + +
Cocl2 - - + -
Nutlin3 - - - +
B
p53 expression
si-p53 - + + +
Cocl2 - - + -
Nutlin3 - - - +
C
DDIT4 expression
Ctrl
shDDIT4
D
DDIT4 expression (Log2)
16384
4096
1024
256
64
16
4
1
Ctrl
DDIT+
E
HIF1α expression
Ctrl
siDDIT4
F
Nanog expression
Ctrl
D1
D4
G
Runx2
ALP
PPARγ
CEBPα
mRNA expression
Ctrl
shDDIT4

S2. Effects of *HIF1α*, *p53* and *DDIT4* knock-down and DDIT4 overexpression. (A) Expression of *HIF1α* after siRNA knock-down of *HIF1α*. (B) Expression of *p53* after siRNA knock-down of *p53*. (C) Expression of *DDIT4* after shRNA knock-down of *DDIT4*. (D) Expression of *DDIT4* after overexpression of DDIT4. (E) Expression of *HIF1α* after siRNA knock-down of *DDIT4*. (F) Expression of *Nanog* in response to hypoxia mimic. MSC were treated with 100μM of CoCl2 for 1 and 4 days and expression of *Nanog* was determined by qRT-PCR. (G) Expression of genes involved osteogenic and adipogenic lineage commitment in undifferentiated MSC and in *DDIT4* depleted cells. All data is from at least three separate experiments.

S3

A

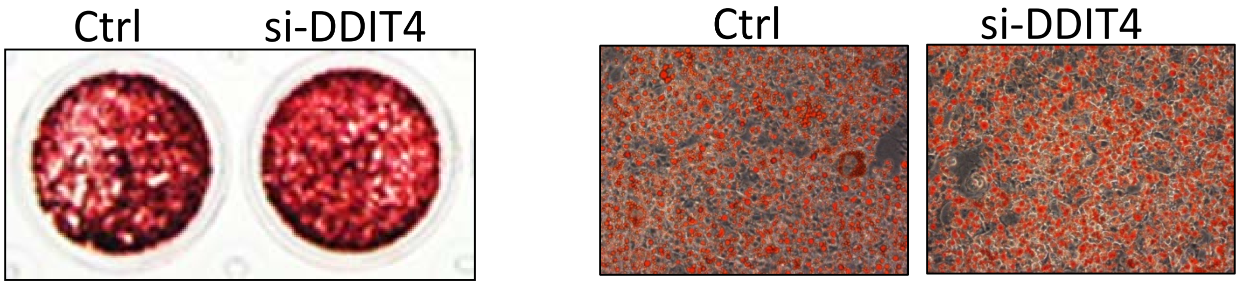
Ctrl
si-DDIT4
Ctrl
si-DDIT4

B

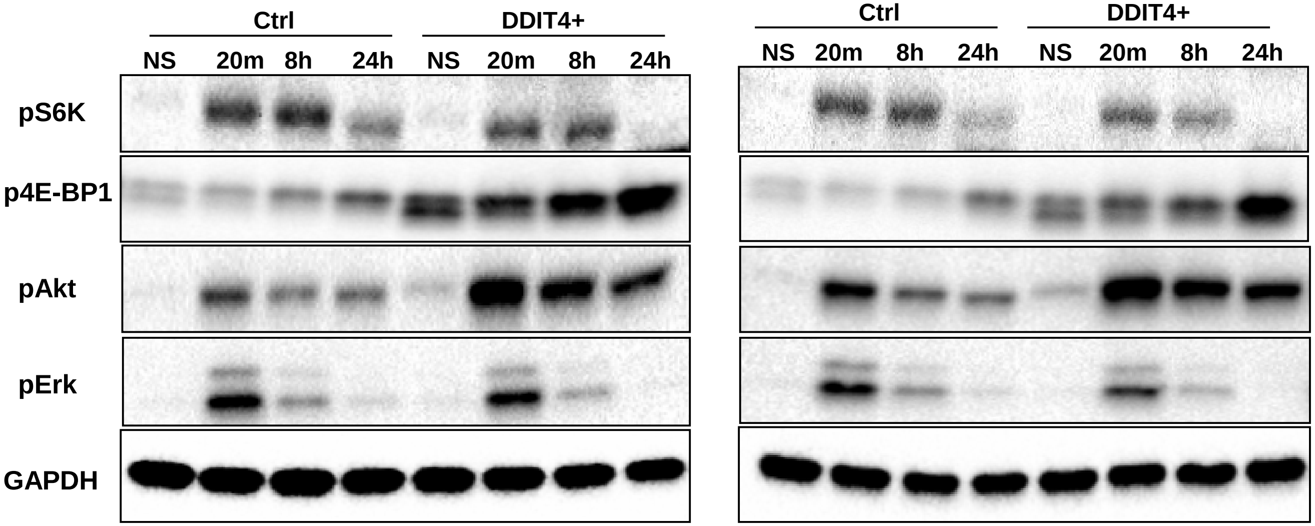
Ctrl
DDIT4+
NS
20m
8h
24h
NS
20m
8h
24h
pS6K
p4E-BP1
pAkt
pErk
GAPDH
Ctrl
DDIT4+
NS
20m
8h
24h
NS
20m
8h
24h

S3. Effects of *DDIT4* knock-down and overexpression in 7F2 cells. (A) Differentiation capacity of 7F2 cells in *DDIT4* depleted cells. (Left) Osteogenic differentiation was assessed by Alizarin Red staining of mineralized matrix and (right) adipogenic differentiation was analyzed following Oil Red O staining. (B) mTOR activity during differentiation of control and cell overexpressing DDIT4. Cells were serum starved for 6 hours and phosphorylation of 44/42 MAPK (Erk1/2), Akt, S6K, and 4E-BP1were analyzed in response to stimulation by (left) osteogenic and (right) adipogenic media for 20 minutes, 6 and 24 hours using Western blotting. Data shown is representative of two separate experiments.

Table S1.

**Human Genes**	**Forward (5` to 3`)**	**Reverse (5` to 3`)**
ALP	AACACCACCCAGGGGAAC	TGGCATGGTTCACTCTCGT
DDIT4	CGAGTCCCTGGACAGCAG	GGTCACTGAGCAGCTCGAAG
FAB-4	GGCCAGGAATTTGACGAAGT	TTTCCATCCCATTTCTGCAC
HIF1α	GCCGAGGAAGAACTATGAACA	CACTGAGGTTGGTTACTGTTGG
KLF4	TTCTCCACGTTCGCGTCT	CGCTTCATGTGGGAGAGC
LPL	CTTCGCCATTCAGAAGATCAG	TGCCTTTCCTTTCTGCAAAT
Nanog	CCAACATCCTGAACCTCAGC	GCTATTCTTCGGCCAGTTGT
Oct-4	GTGGAGGAAGCTGACAACAA	CACTCGGTTCTCGATACTGG
OPN	GCCGAGGTGATAGTGTGGTT	TGAGGTGATGTCCTCGTCTG
p53	GATGGAGAATATTTCACCCTTCA	CTGAGTCAGGCCCTTCTGTC
PPAR-γ	TGCAGTGGGGATGTCTCATA	CAGCTGGTCGATATCACTGGA
RPL13A	GGATGGTGGTTCCTGCTG	TGGTACTTCCAGCCAACCTC
Runx-2	AATGGTTAATCTCCGCAGGTC	TTCAGATAGAACTTGTACCCTCTGTT

Table S2.

**Mouse**	**Forward (5` to**	**Reverse (5` to**
ALP	CAAGGACATCGCATATCAG	TCCACATCAGTTCTGTTCTTC
CEBP?	GAGGGGAGGGACTTAGGTG	TAGGTGGAGGTGCAAA AGC
LPL	AGACCTTCGTGGTGATCCA	CATTGGAGTCAGGTTCTCTCTT
Osc	GCTGCGCTCTGTCTCTCT	TTCACTACCTTATTGCCCTCC
PPAR-?	TCACAAGAGCTGACCCAAT	AATAAGGTGGAGATGCAGGTT
RPL13A	CCTGCTGCTCTCAAGGTTG	GGCTGTCACTGCCTGGTAC
Runx-2	TACCTGCCATCACTGACGT	CTGCCTGGCTCTTCTTACTG

Table S1&2, Related to Supplemental Experimental Procedures; Primer sequences. Product sizes were between 98-102 bp.

SUPPLEMENTAL EXPERIMENTAL PROCEDURES

Cell culture

Primary human MSCs from young male and female adults (aged 21-25) were purchased from Lonza (Slough, UK) and characterized as shown previously (Gharibi and Hughes, 2012). 7F2 (ATCC; LGC Standards) immortalized murine progenitor cell line was a kind gift from Dr Bronwen Evans. Cells were cultured in α-Minimal Essential Medium (MEM), penicillin (50U/ml), streptomycin (50μg/ml) (all from Sigma-Aldrich, Poole, Dorset, UK), Glutamax (2mM) (Life Technologies, Paisley,UK) and 10% fetal bovine serum (FBS) (Sigma–Aldrich) and maintained at 37°C in a humidified 5% CO_2:95% air atmosphere. For differentiation of MSCs to osteoblasts, cell were seeded at a density of 10,000 cell/cm^2 and induced with growth medium supplemented with 0.1μM dexamethasone, 0.05mM L-ascorbic acid 2-phosphate (AA) and 10mM β-glycerophosphate (Sigma–Aldrich). Adipogenic differentiation was induced at a density of 40,000 cell/cm^2 with growth medium supplemented with, 1μM dexamethasone, 0.25mM isobutylmethylxanthine, 50μM indomethacin and 10μg/ml insulin (Sigma–Aldrich). For differentiation of 7F2, cell were seeded at 5000 cell/cm^2, osteogenesis was induced in presence of 50 μg/ml AA, 0.01μM dexamethasone and 5mM β-glycerophosphate and adipogenesis was induced by the addition of 50 μg/ml (AA), 0.1μM dexamethasone and 50 μM indomethacin.To assess osteogenic differentiation, RNA was isolated after 14 days of incubation and mRNA expression of markers of differentiation i.e. *Runx2*, *alkaline phosphatase (ALP)*, *Osteocalcin* (*Osc*) or *osteopontin* (*Opn*) was determined by quantitative (q)RT-PCR and accumulation of calcium deposits was visualised and quantified by staining with Alizarin Red dye as described previously (Gharibi et al., 2011). Adipogenesis was assessed by qRT-PCR analysis of markers of differentiation i.e. *PPARγ*, CEBPα, *fatty acid binding protein* (*FAB*)4 and *lipoprotein lipase* (*LPL*) and lipid accumulation was visualised and quantified following staining with Oil red O dye as described previously(Gharibi et al., 2011).

Production of single cell clones

To achieve single cell clones from subpopulations of MSCs, cell were diluted to 1 cell per 100μl per well of 96 well plates. The presence of single cell in each well was evaluated using a phase-contrast microscope, and wells with more than one cell were excluded from the study. MSC single clones were subsequently expanded and assessed for differentiation capability and subdivided into 3 categories; clones with high osteogenic potential, high adipogenic potential, or with low differentiation

potential. Subsequently three clones from each category were chosen to be analysed for gene expression by microarray.

Microarray analysis

Total RNA was extracted using TRI reagent (Life Technologies) and Phase Lock Gel Heavy tubes (5 Prime, VWR, Leicestershire, U.K.) according to the manufacturer's instructions. RNA purity and quantity was assessed by nanodrop (Fisher Scientific) and Qubit 2.0 (Life Technologies). RNA integrity was determined using an Agilent 2100 Bioanalyzer (Agilent Technologies, Cheshire, UK). The gene expression analysis was performed using the Illumina whole-genome expression array HumanHT-12 v4.0 Expression BeadChip according to the manufacturer's instructions. Briefly, 500 ng of total RNA was processed and cRNA was hybridized to an Illumina whole genome expression chip. The Beadchips were scanned on the Illumina iScan System with iScan software. The raw data were processed for the background signal, normalized, and differences in gene expression were compared by the Differential Expression Algorithm using the Illumina custom error model of GenomeStudio software (Illumina, Inc.). Genes with a Diffscore of ±13, equivalent to $p<0.05$, were considered as being significantly down- or upregulated; a DiffScore of ±20, ±30 or ±40 was equivalent to a *p*-value of <0.01, <0.001, or <0.0001, respectively.

Live cell RNA sorting

Cell were sorted based on *DDIT4* RNA expression level using novel SmartFlare™ RNA detection probes (SmartFlares; Millipore, Watford, UK). MSCs were cultured at 80-85% confluence for 24h prior to smartflare treatment and subsequently incubated for 16h with *DDIT4* specific custom probe (DDIT4-2 Hu-Cy5 (SFC-456) with sequence: CTGACGCTGAGCACTGGCTTCCGAGTC) or Scramble-Cy5 control SmartFlare probe at a 1:1000 dilution. Cells were sorted using a FACSAria (Becton Dickinson Biosciences, San Jose, CA) into two population of low and high (gated lowest or highest 15% population based on APC fluorescence intensity) based on *DDIT4* expression level. Scramble-Cy5 control was used for detecting unspecific targeting and background signals. Sorted sub-populations were then returned to cell culture and expanded for further analysis.

Stable and transient transfection

Short hairpin (shRNA) plasmids for stable knockdown of *DDIT4* and negative control were obtained from Qiagen (Qiagen, West Sussex, UK). Initially 4 shRNA were tested and subsequently one was selected for further studies. shRNA sequence for DDIT4

was (shDDIT4) AGCCAGGTGGGCAAAGAACTA and negative control for nonspecific effects was (NC) ggaatctcattcgatgcatac (catalog no. 336313KH1172N). The plasmids were transformed into bacteria and purified using EndoFree Plasmid purification Kit (Qiagen) according to manufacturer's instruction. The transient knockdown of *DDIT4*, *HIF1α* and *p53* genes were carried out using siRNA from Qiagen comprising the following sequences; siDDIT4, CGGCAGGACGCACTTGTCTTA; siHIF1α, AGGAAGAACTATGAACATAAA; sip53, AAGGAAATTTGCGTGTGGAGT. For overexpression studies full-length *DDIT4* construct was purchased from Life Technologies. The construct was cloned into pcDNA-DEST47 gateway expression vector (Life Technologies), transformed into bacteria and purified according to manufacturer's instruction. pcDNA/GW-47/CAT was used as control. Transfection was performed using the Neon transfection system (Life Technologies) according to the manufacturer's instructions. Briefly, cells were suspended in R buffer, mixed with DNA construct, shRNA plasmid, siRNA or controls and subjected to electroporation. Cells were subsequently incubated for 24h at 37°C in a humidified 5% CO_2:95% air atmosphere prior to further investigation.For stable expression, transfected cells were selected with G418 sulphate (0.4 mg ml^{-1}) (Life Technologies) and expanded before experimentation. Validation of transfection efficiencies is shown in figure S3A-D.

Western blotting analysis

Following stimulation, cells were washed in ice-cold PBS containing 1 mM sodium orthovanadate and lysed in ice-cold radioimmunoprecipitation assay buffer [50 mM tris(hydroxymethyl)aminomethane (Tris)-hydrochloric acid (HCl), pH 7.5, 150 mM sodium chloride (NaCl), 1% Nonidet P-40, 0.1% Sodium dodecyl sulphate (SDS), 0.5% sodium deoxycholate] containing a protease inhibitor cocktail (Sigma–Aldrich),1 mM sodium orthovanadate and 0.1 mg/ml phenylmethylsulfonyl fluoride. Cell lysates and biotinylated protein ladder (Cell Signalling, New England Biolabs, Hitchin, UK) were mixed with Laemmli buffer (Bio-Rad, Hempstead, UK) and subjected to SDS-PAGE. Proteins were transferred onto PVDF membranes and incubated overnight at 4°C with primary antibodies against DDIT4 (Proteintech), phospho-Akt (Ser473), phospho-p44/42 MAPK (Erk1/2) (Thr202/Tyr204), phospho- p70 S6 Kinase (Thr389), phospho-p70 S6 ,phospho-4E-BP1 (Thr37/46) eIF4E (Cell Signalling, New England Biolabs) and GAPDH (Sigma–Aldrich). Secondary antibodies (1:2000) conjugated to horseradish peroxidase were then applied for 1 hour at room temperature, and proteins visualized and photographed using ECL Prime detection reagent (GE Healthcare, Bucks, UK) and Molecular Imager Gel Doc XR+ documentation and analysis System with Image Lab Software (Bio-Rad).

qRT-PCR analysis

Total RNA was extracted using TRI reagent (Life Technologies) and Phase Lock Gel Heavy tubes (5 prime) according to the manufacturer's instructions. RNA purity and quantity was assessed by nanodrop (Fisher Scientific) (A_{260}/A_{280} 1.8-2 was considered suitable for further analysis), possible contaminating DNA was removed and cDNA prepared from 1 µg RNA using QuantiTect Reverse Transcription Kit (Qiagen) according to the manufacturer's instructions. qRT-PCR was performed on a Mx3000P real time PCR system (Agilent Technologies) using iTaq SYBR Green qPCR master mix (Bio-Rad) and primer pairs as listed in tables (SI 1&2). PCR conditions consisted of 1 cycle of 95 °C for 40 sec and 40 cycles of 95 °C for 5 sec and 60 °C for 20 sec. RPL13a was used as an invariant housekeeping gene.

Colony Forming Unit (CFU) assay

MSCs were seeded at a density of 400cell/well in 6 well plates and cultured for up to14 days with media replenished every 3-4 days. Cells were washed with PBS, fixed for 15 min with 4% formaldehyde in PBS, stained for 30 min with 0.5% Crystal Violet and washed with PBS.

Cell proliferation assay

Cell number was counted at each passage using a haemocytometer and subcultured for further expansion. Cell proliferation (DNA synthesis) was assessed by measuring 5-ethynyl-2′-deoxyuridine (EdU) DNA incorporation using the Click-iT EdU Alexa Fluor 647 cell proliferation assay kit (Life Technologies). Briefly, cells were treated with EdU at 10 µg/ml for 48 hours, harvested by trypsinization, washed in PBS/1% BSA, and fixed with Click-iT fixative. The cells were then permeabilized using saponin-based permeabilization reagent, treated with the Click-iT EdU reaction cocktail in the dark, and washed with saponin-based permeabilization reagent. The number of EdU-positive cells was determined using a FACS-Canto II flow cytometer, and data analysis was performed using DIVA software (Becton Dickinson Biosciences).

Immunostaining

For immunohistochemistry femurs of 5 weeks old CD-1 wild type mice were dissected and cleaned of surrounding tissue. The bones were then fixed in 4% formaldehyde in PBS for 3 days and decalcified in 10% EDTA for further 7 days. Samples were dehydrated and embedded in paraffin and sectioned. Paraffin sections were stained with goat-anti-LepR-biotin (R&D system, Abingdon, UK) and rabbit anti-DDIT4

(Proteintech) antibodies and images were acquired with a phase-contrast microscope.

Data Analysis

Statistical comparisons of means were made by Student's *t* test (IBM, SPSS 21) when comparison was between two groups and by one-way ANOVA (SPSS 21) and post hoc analyses using the Tukey test to evaluate the differences among the mean values between groups. A *p*-value of less than .05 was considered statistically significant.

SUPPLEMENTAL REFERENCES

Gharibi, B., Abraham, A.A., Ham, J., and Evans, B.A. (2011). Adenosine receptor subtype expression and activation influence the differentiation of mesenchymal stem cells to osteoblasts and adipocytes. Journal of Bone and Mineral Research *26*, 2112-2124.

Gharibi, B., and Hughes, F.J. (2012). Effects of Medium Supplements on Proliferation, Differentiation Potential, and In Vitro Expansion of Mesenchymal Stem Cells. Stem Cells Translational Medicine *1*, 771-7
